# ET_B _receptor polymorphism is associated with airway obstruction

**DOI:** 10.1186/1471-2466-7-5

**Published:** 2007-04-30

**Authors:** Camille Taillé, Armelle Guénégou, Abdelhamid Almolki, Marie Piperaud, Bénédicte Leynaert, Sandrine Vuillaumier, Françoise Neukirch, Jorge Boczkowski, Michel Aubier, Joëlle Benessiano, Bruno Crestani

**Affiliations:** 1 Service de Pneumologie, Hôpital Bichat-Claude Bernard, Assistance Publique-Hôpitaux de Paris 75018 Paris, France; 2 Institut National de la Santé et de la Recherche Médicale (INSERM) U700, Faculté de Médecine Xavier Bichat, 75018 Paris, France; 3 Service de Biochimie, Hôpital Bichat-Claude Bernard, Assistance Publique-Hôpitaux de Paris, 75018 Paris, France; 4 Centre de Ressources Biologiques – Centre d'Investigation Clinique, Hôpital Bichat-Claude Bernard, Assistance Publique-Hôpitaux de Paris, 75018 Paris, France

## Abstract

**Background:**

Endothelin-1 (EDN1) has been involved in the development of airway obstruction and inflammation in asthma. Several polymorphisms have been identified among the genes encoding for preproET1, an inactive precursor of ET-1, and for ET_A _(EDNRA) and ET_B _(EDNRB), the two receptors for EDN1. In the present work, we hypothesised that molecular variation in these genes could be a major determinant of the degree of bronchial obstruction. The purpose of this study was to investigate whether the genetic polymorphisms of *preproET-1*, *EDNRA *and *EDNRB *genes were associated with the degree of airway obstruction, assessed by FEV_1_.

**Methods:**

Polymorphisms of *preproET-1, EDNRA and EDN*RB were first studied in a population of adult asthmatic patients. Results were confirmed in a large population of adults from the general population from the ECRHS II study.

**Results:**

In our population of adult asthmatic patients, the EDNRB-30G>A (Leu277Leu) polymorphism (GG genotype) is strongly associated with a low FEV_1 _and with a higher percentage of patients with FEV1 < 80% of predicted value. No relationship was found between pulmonary function and EDNRA-1363C>T (His323His) or preproET-1-595G>T (Lys198Asp) polymorphism. In the adult population from the ECRHS II, we found a similar association between GG genotype and a low FEV_1 _or a higher percentage of subjects with FEV1 < 80% predicted, especially in the subgroups of asthmatics subjects (OR = 4.31 (95%CI 1.03 – 18.04)) and smokers (OR = 7.42 (95%CI 1.69 – 32.6)).

**Conclusion:**

the EDNRB-30G>A polymorphism could be a determinant of airway obstruction in humans with predisposing factors such as tobacco smoke exposure or asthma.

## Background

Asthma is a complex disorder characterised by airway hyperresponsiveness and airway inflammation [1]. The age-related rate of decline in lung function is greater in adults with asthma than in those without asthma [2,3] and is the result of chronic airway inflammation and airway remodelling [4]. The precise mechanisms underlying the remodelling process are not totally understood [5] but a genetic susceptibility has been suspected since all subjects with asthma do not deteriorate their lung function [2].

Endothelin-1 (EDN1) is a 21 amino acid peptide synthesized from preproendothelin-1 (preproET-1), an inactive precursor peptide encoded by a specific gene. PreproET-1 is cleaved by an endopeptidase to the intermediate form namely "big-endothelin", then cleaved by the endothelin converting enzyme to the mature form EDN1 [6]. There are several lines of evidence suggesting that EDN1 may play a role in the development of chronic airway obstruction in asthma [7,8]. EDN1 is strongly expressed in the lung and is synthesised by many different cells including epithelial cells, inflammatory cells and smooth muscle cells [6,9,10]. Increased concentrations of EDN1 have been found in the bronchial lavage of asthmatics [11], and overexpression of EDN1 in the airways of asthmatic patients has been demonstrated [12]. EDN1 may act as a paracrine hormone causing sustained contraction of airway smooth muscle cells [13,14], accelerated growth of both fibroblasts and airway smooth muscle cells [15,16] and increased oedema formation and mucus secretion [8], contributing to airway obstruction. EDN1 is implicated in the initiation of eosinophilic airway inflammation [17]. Furthermore, chronic overexpression of human EDN1 has been shown to induce peribronchial fibrosis in transgenic mice [18].

The biological effects of EDN1 are mediated by the interaction of EDN1 with two specific receptors termed EDNRA and EDNRB. Both receptors are present in the human bronchial airways [19,20] but EDNRB is predominant [19]. EDNRA has been involved in EDN1 induced prostanoid and nitric oxide release and airway smooth muscle proliferation [21], whereas EDNRB mediates the contractile effect of EDN1 [19].

The genomic structure of human *preproET-1, EDNRA and EDNRB *have been determined and several polymorphisms have been identified among these genes [22,23]. As suggested by previous studies showing an association between EDN1 polymorphism and asthma [24] or atopy [25], we hypothesised that molecular variations in theses genes could be a major determinant of the degree of bronchial obstruction in asthmatic patients. The purpose of this study was therefore to investigate whether genetic polymorphisms of *preproET-1*, *EDNRA *and *EDNRB *genes were associated with the degree of airway obstruction assessed by the forced expiratory volume in one second (FEV_1_). We chose to concentrate our project on polymorphisms for which heterozygotes are frequent : 1/the G to T substitution located in exon 5 at position 595 of the preproET-1 gene (preproET-1-595G>T)(rs5370) [22], 2/the C to T substitution located in exon 8 at position 1363 of the EDNRA gene (EDNRA-1363C>T)(rs5333) [23] and 3/the G to A substitution located in exon 4 at position 30 of the EDNRB gene (EDNRB-30G>A)(rs5351) [23]. We first genotyped a population of 162 adult asthmatic patients recruited from our outpatient clinic and found a strong association between the polymorphism of *EDNRB *and FEV_1_. No association was found with the *EDNRA *or the *preproET-1 *genes. To test the reproducibility of this association observed between *EDNRB *and FEV1, we performed a similar analysis, focusing on the *EDNRB *polymorphism, on a larger group of adult subjects recruited from the general population as part of the European Community Respiratory Health Survey (ECRHS II).

## Methods

### 1. Hospital-based population of asthmatic patients

We studied 162 consecutive adult patients with asthma, evaluated in the outpatient clinic of Bichat University Hospital. Asthma was diagnosed on clinical grounds according to the American Thoracic Society official statement [26]. All patients were Caucasians, born in France, and gave informed consent to participate in the study. This study was approved by the ethical committee of Bichat-Paris Nord.

A flow-volume curve was performed in all patients in a stable condition at least one month after an acute exacerbation of asthma. The flow-volume curve was performed according to the European Respiratory Society guidelines [27]. The patients did not receive any bronchodilators within 12 hours before the procedure. We analysed the pre-bronchodilator FEV_1 _and the FEV_1_/FVC ratio.

### 2. Population-based sample of subjects recruited as part of the ECRHS

The European Community Respiratory Health Survey (ECRHS) II is a multi-centre, international study in which representative community based samples of young adults have their lung function assessed on two occasions about 9 years apart. The methods for ECRHS I and ECRHS II have been published in more detail elsewhere [28,29] and further information is available from the study website [30]. In ECRHS II, blood samples were taken in order to extract DNA.

All subjects recruited in the centres of Grenoble, Montpellier and Paris (France), born in France, for whom spirometry data for both ECRHS I and ECRHS II and blood samples were available, were eligible for this study. The sex-ratio did not differ between the 653 eligible and 732 ineligible subjects (including 493 subjects lost to follow-up, 220 without blood sample and 19 with blood sample but unsatisfactory spirometry). Eligible subjects were slightly older and more likely to be non-smokers than ineligible subjects (data not shown). For the analyses, we included the eligible subjects who were genotyped for the *EDNRB *polymorphism (n = 632) : 21 subjects were excluded as the genotype was not available; they did not differ from the other eligible subjects in terms of age, sex-ratio, smoking status, and FEV1. Subjects who answered positively to the question "Have you ever had asthma ?" were considered as asthmatics.

Written informed consent was obtained from each subject before inclusion and the protocol was approved by the French Ethics Committee for Human Research and by the National Committee for Data Processing and Freedom.

### 3. Determination of preproET-1, EDNRA and EDNRB genotypes

Each genetic polymorphism was studied from genomic DNA that was obtained from blood leukocytes, using standard phenol extraction as previously described [31]. Genotyping of these polymorphisms was conducted using an adapted method of DNA amplification by polymerase chain reaction (PCR) procedure, with specific primers. PCR products were digested by specific restriction enzymes and separated by appropriate agarose gel electrophoresis.

#### • PreproET-1 gene polymorphism

The *preproET-1 *gene is located on chromosome 6p23-24. The G to T transition at nucleotide 595 in exon 5 abolishes one Ban II restriction site. Amplification of a 320-bp fragment was performed using specific primers (5'-ATGATCCCAAGCTGAAAGGCG-3' and 5' GCTGAGAGGTCCATTGTCATCC-3'). After initial denaturation at 94°C for 5 min., amplification was performed in a DNA thermocycler (Gene Amp PCR System, Perkin Elmer ; denaturation : 40 s at 94°C, annealing: 30s at 55°C, extension : 40 s at 72°C, 35 cycles). The genotypic polymorphism was defined as GG (wild homozygote), GT (heterozygote) and TT (mutated homozygote).

#### • EDNRA gene polymorphism

The *EDNRA *gene located in the short arm of the chromosome 4 presents a C to T substitution located in exon 8 at 1363 position. Amplification of a 297-bp fragment using specific mutagenic primers (5'-GAAGTCTAAAACACACCTAAGA-3' and 5'-TAGGTTCATACTGAAAACCCTAA-3' underlined nucleotide represents the mutated nucleotide), introduces a DdeI restriction site on the normal C allele which is lost on the mutated T allele. After initial denaturation at 94°C for 5 min, amplification was performed (denaturation : 40 s at 94°C, annealing: 30s at 50°C, extension: 40 s at 72°C, 35 cycles). The genotypic polymorphism was defined as CC (wild homozygote), CT (heterozygote) and TT (mutated homozygote).

#### • EDNRB gene polymorphism

The *EDNRB *gene is located on the chromosome 13q22 and presents a G to A substitution located in exon 4 at position 30. Amplification of a 147-bp fragment using specific mutagenic primers (5'-GACAGCAAAAGATTGGTGGAT-3' and 5'-CTTACCTGCTTTAGGTATC-3'), introduces a Fok I restriction site on the normal G allele which is abolished on the A allele. After initial denaturation at 94°C for 5 min, amplification was performed (denaturation : 30 s at 94°C, annealing: 40s at 46°C, extension: 40 s at 72°C, 35 cycles). The genotypic polymorphism was defined as GG (wild homozygote), GA (heterozygote) and AA (mutated homozygote).

The results of genotyping of 200 patients were consistent with the nucleotide sequence of exons 1–7 previously determined on an automated sequencer.

### 4. Relative quantification of mRNA transcribed from each allele of the ET_B _receptor gene

In order to test the hypothesis that the EDNRB-30G>A polymorphism could alter EDNRB mRNA level, we quantified the mRNAs transcribed from each allele of the gene. For that purpose, we analysed lung cDNA from 12 heterozygote patients for the ET_B_/G30A polymorphism. Lung samples were obtained at the time of surgery for lung cancer, in a macroscopically normal area.

Total mRNA of heterozygote patients was extracted from homogenized lung tissues using TRIZOL reagent (Life Technologies, France). For cDNA synthesis, reverse transcription was performed by use of random hexamer (50 ng/μl) (Pharmacia, Guyancourt, France) and 10 U/μl Superscript II (Gibco France) in presence of 1 μg of RNA, 0.5 mM dNTPs (Pharmacia, Guyancourt, France), 10 mM dithiothreitol, 0.1 μg/μl BSA and 1U/μl of Rnasine (Promega, Charbonnières, France). The mixture was incubated at 42°C for 60 minutes. Polymerase chain reaction was used to amplify cDNAs with specific primers : E3S : 5'-TTG CTT CAT CCC GTT CAG A-3' and E4AS 5' biotin-CAC CTT TTC TTT CTC AAC AT-3'. The extension assay was performed by the dideoxy chain termination method with a fluorescent primer (5'fluorescein-AAG ACA GCA AAA GAT TGG TGG-3') finishing at two bases from the G30A polymorphism according to a previously described procedure [31,32]. The substitution of dATP by ddATP in the termination mixture resulted in the synthesis of allele-specific extension products of 24 bases and 28 bases for A and G alleles respectively. After migration on an automated sequencer (ABI PRISM 310, Applied Biosystem, France), both products were detected as fluorescent peaks. The ratio of area under the curve of both detected peaks from each sample of heterozygote patient was determined with the Genescan analysis software (Applied Biosystem, France). The ratio of area under the curve for the two peaks was close to 1 when genomic DNAs from heterozygote patients was used.

### 5. Sequence analysis of ET_B _cDNA from lung tissues

In order to test for a possible existence of other mutations of the *EDNRB *gene in linkage disequilibrium with the EDNRB-30G>A polymorphism, the nucleotide sequence of exons 1–7 of lung cDNA obtained from 10 heterozygote patients was determined on an automated sequencer. The entire coding region was obtained in two overlapping amplification products on cDNA with the following primers: ET_B _1 bis 5'-ACC GGA CGC CTT CTG GAG CA-3' and ET_B _3AS 5'-ACA ATT TCT ACT GCT CAT-3', ET_B _2S 5'-GAG CTG TTG CTT CTT GGA GTA-3' and ET_B _7AS 5'-AAT GAC TTC GGT CCA ATA TAA-3'.

### 6. Statistical analysis

#### • Asthmatic population

Allele frequencies and genotypes repartition were counted. Functional respiratory parameters (FEV1 as % of predicted value and FEV1/FVC ratio) are expressed as mean value ± SD. Differences between the studied groups defined according to the genotypes were evaluated by 2-way analysis of variance (ANOVA); the significance level was fixed at 5% (p values less than 0.05).

#### • ECRHS II population

For the EDNRB, we either analysed the 3 genotypes separately or pooled genotypes AA and AG compared to genotype GG. FEV1 as %predicted and FEV1/FVC ratio were considered as continuous variables, and analysed using ANOVA. Subjects with FEV1 %predicted lower or equal to 80% were considered to have airway obstruction. Statistical differences in the frequency of airway obstruction were tested using Chi-square tests, or Fisher Exact test when the numbers were too low. This outcome was adjusted for confounders in binary logistic regressions. Confounding factors included sex, centre and smoking status (coded in 4 classes: never-smokers, ex-smokers (>1 year), moderate and heavy smokers (smokers and ex smokers < 1 yr, with < 20 cig/day and ≥ 20 cig/day, respectively). To make sure of the lack of stratification in the underlying population for the polymorphism, Hardy-Weinberg structure was tested. All statistics were computed with SAS software package (SAS Institute, Cary NC, USA).

#### • Lung mRNA transcription

The ratio of area under the curves of both detected peaks obtained from cDNA was compared to the ratio obtained from genomic DNA of heterozygote patients. Data were expressed as mean + standard deviation and analyzed using t test for paired data. Significance for all statistics was accepted at P < 0.05.

## Results

### 1. Lung function and genotype in asthmatic patients

#### • Pulmonary function tests and frequencies of genotypes in asthmatic patients

Patients were 86 women and 76 men, with a mean age of 46.3 (± 16 years). All were never smokers; 30.8% had total serum IgE levels higher than 100 UI/ml. In the whole population, mean FEV_1 _amounted to 73.7 ± 21.20 % of predicted values and mean FEV_1_/FVC ratio was 65.5 ± 15.5 %. The distribution of genotypes for each polymorphism is presented in table 1 and was similar to previoulsy reported data in the general population (21, 22). *EDNRB *polymorphism displayed a Hardy-Weinberg structure.

**Table 1:** Frequencies of genotypes and alleles for each variants among 162 asthmatic patients

**Genotypes frequencies**		**PreproET-1**		**EDNRA**		**EDNRB**
		n (%)		n (%)		n (%)

	**GG**	101 (62.3%)	**CC**	80 (49.4%)	**GG**	64 (39.5%)
	**GT**	55 (34%)	**CT**	67 (41.3%)	**GA**	75 (46.3%)
	**TT**	6 (3.3%)	**TT**	15 (9.3%)	**AA**	23 (14.2%)
**Allele frequencies**						

	**G**	0.79	**C**	0.70	**G**	0.63
	**T**	0.21	**T**	0.30	**A**	0.37

#### • Relationship between genotypes, FEV_1 _and FEV_1_/FVC in asthmatic patients

The *preproET-1 *gene and the *EDNRA *gene polymorphisms did not correlate with any of the pulmonary functional parameters studied (data not shown). By contrast, the *EDNRB *gene polymorphism was significantly related to the FEV_1 _value (p = 0.0039). Indeed, mean FEV_1 _was lower in the homozygotes GG (68.7 ± 19.2%) than in patients with one or more allele A (76.9 ± 21.9%, p = 0.016) (table 2). FEV_1 _tended to be lower in heterozygotes GA than in mutated homozygotes AA, without reaching statistical significance. The FEV_1_/FVC ratio was similar in the different groups. However, FEV_1_/FVC ratio tended to be lower (p = 0.086) and the percentage of patients with airway obstruction tended to be higher (p = 0.09) in homozygotes GG than in AA+AG patients (table 2).

**Table 2:** Differences in lung function measurements and prevalence of airway obstruction according to the *EDNRB *genotype in the hospital-based population of asthmatic patients.

	**FEV**_1 _(%predicted)	**FEV**_1_**/FVC**	**FEV1= 80%pred**
	mean (SD)	mean (SD)	% (n)
	
**AA (n = 23)**	85.65 (22.6)	67.65 (14.7)	43.5 (10)
**AG (n = 75)**	74.2 (21.2)	67 (15.7)	61.3 (46)
**GG (n = 64)**	68.75 (19.2)	62.82 (15.8)	78.1 (45)
***p***	***0.0039***	***0.22***	***0.072***

**AG+AA (n = 98)**	76.9 (21.9)	67.15 (15.4)	57.42(56)
**GG (n = 64)**	68.7 (19.2)	62.8 (15.8)	70.3 (45)
***p***	***0.016***	***0.086***	***0.09***

Although uneven distribution of the sex ratio in the different genotypes could participate in the higher FEV_1 _measured in mutated homozygotes (27), this variable was not different according to the *EDNRB *genotype (AA :10 women, 13 men, GA : 44 women, 31 men and GG : 32 women, 32 men, p = 0.36). Moreover, the mean age of the patients did not differ in relation to the *EDNRB *genotype (AA: 42 ± 15 years, GA : 50 ± 17 years and GG: 48 ± 15 years, p = 0.2).

### 2. Results from ECRHS

In ECRHS II, the subjects were aged 44.3 ± 7.2 yr and approximately half of the subjects were men. Forty-three percent were never-smokers, 29.7% were ex-smokers, 18.7% were moderate smokers (< 20 cig/day) and 8.6% were heavy smokers (≥ 20 cig/day). As expected in this young adult population, subjects showed normal FEV1 values (105.7 ± 14.3 %predicted). Among the 632 French subjects included, only 4.3% (n = 27) had airway obstruction as defined by a FEV1 = 80%predicted. Genotypic distribution in the 3 centres is shown in Table 3. Overall, genotypes AA, AG and GG accounted for 17.3%, 48.1% and 34.7%, respectively. *EDNRB *polymorphism displayed a Hardy-Weinberg structure in all centres, as well as following pooling of all centres.

**Table 3:** Frequencies of genotypes and alleles for EDNRB-30G>A among the ECRHS population

	**Grenoble (n = 232)**	**Montpellier (n = 128)**	**Paris (n = 272)**	**Total (n = 632)**
Genotype, %				

AA	16.8	21.9	15.4	17.3
AG	47.0	46.9	49.6	48.1
GG	36.2	31.3	34.9	34.7

Allele, %				

A	40.3	45.3	40.3	41.3
G	59.7	54.7	59.7	58.7

The prevalence of airway obstruction (FEV1 = 80%predicted) was higher among subjects genotyped GG than in those genotyped AA or AG. In multivariate logistic regression, the risk of airway obstruction was not different between centres or between men and women. Heavy smokers had a four-fold increased risk compared to non-smokers (adjusted Odds-Ratio (OR) = 4.04 (95% Confidence Interval (CI) 1.22–13.43). When pooling together subjects with AA and AG genotypes, and after adjustment for centre and smoking, the risk of airway obstruction was higher in the subjects carrying the GG genotype, as compared to other subjects (OR = 3.19 (95%CI 1.43 – 7.15)). When the analysis was run separately in never smokers to discard any possible residual confounding by tobacco, there was a tendency for a higher frequency of airway obstruction in GG subjects as compared to AA+GA (7.1% vs. 0.6%, respectively, p = 0.004 using Fisher's exact test). However the prevalence of airway obstruction was very low in this group of subjects (2.97%, overall), and no association was observed for FEV1 %predicted or FEV1/CVF ratio. Only 54 subjects were considered as heavy smokers at the time of the survey. In order to assess the association in subjects at risk of airway obstruction because of tobacco smoking, we analysed separately the 201 subjects who reported a cumulative history of smoking of more than 12 pack years. When smokers who cumulated more than 12 pack-years were considered, the prevalence of airway obstruction reached 4.97%. In this group, airway obstruction was significantly more frequent in GG subjects (adjusted OR = 7.42 (95%CI 1.69 – 32.6)) and the FEV1 %predicted was lower in carriers of the GG genotype as compared to AA+AG (table 4). Similar results were obtained when the analysis was further restricted to the 118 subjects with a cumulated history of smoking of more than 20 pack years (results not shown).

**Table 4:** Differences in lung function measurements and prevalence of airway obstruction according to the *ETB *genotype in the ECRHS population

	**FEV**_1 _(%predicted)*		**FEV**_**1**_/FVC*		**FEV1 ≤ 80%pred**	
	mean	(SE)	mean	(SE)	%	(n)

**All patients (n = 632)**						
**AA (n = 109)**	104.8	(1.36)	84.2	(0.7)	4.6	(5)
**AG (n = 304)**	107.0	(0.81)	83.4	(0.4)	2.0	(6)
**GG (n = 219)**	104.4	(0.95)	83.2	(0.5)	7.3	(16)
***p***	***0.09***		***0.47***		***0.012***	
**AG+AA (n = 413)**	106.4	(0.70)	83.6	(0.3)	2.7	(11)
**GG (n = 219)**	104.4	(0.96)	83.2	(0.5)	7.3	(16)
***p***	***0.09***		***0.55***		***0.005***	

**Never smokers (n = 269)**						
**AG+AA (n = 170)**	107.4.	(1.1)	84.3	(0.5)	0.6	(1)
**GG (n = 99)**	105.9	(1.4)	83.6	(0.7)	7.1	(7)
***p***	***0.39***		***0.40***		-^†^	

**Smokers ≥ 12 pack-Years (n = 201)**						
**AG+AA (n = 138)**	105.7	(1.2)	81.9	(0.6)	2.2	(3)
**GG (n = 63)**	99.6	(1.8)	82.4	(0.9)	11.1	(7)
***p***	***0.007***		***0.6***		***0.007***	

**Asthmatics (n = 87)**						
**AG+AA (n = 52)**	102.8	(2.4)	81.2	(1.1)	5.8	(3)
**GG (n = 35)**	93.1	(2.9)	77.9	(1.4)	22.9	(8)
***p***	***0.012***		***0.07***		***0.02***	

* adjusted for centre and smoking habits for FEV1%predicted, adjusted for centre, sex and smoking habits for FEV1%CVF.

† Chi-square statistic not valid.

Finally, the analysis was conducted in subjects who reported to have ever had asthma. Similarly to what was observed in the sample of asthmatic patients recruited at the University clinic, there was a tendency for both FEV1%predicted and FEV/FVC ratio to be lower in GG subjects compared to AA+AG subjects (table 4). In subjects with asthma recruited in ECRHS, the prevalence of airway obstruction was 12.6%, and it was more frequent in GG subjects compared to AA+AG (adjusted OR = 4.31 (95%CI 1.03 – 18.04)).

### 3. Relative quantification of ET_B _mRNA transcribed from each allele of the gene

In order to determine whether the EDNRB-30G>A polymorphism modified the expression of the EDNRB gene, we quantified the relative level of EDNRB mRNA for each allele (wild and mutated) in 12 heterozygote patients. For each patient, the ratio of area under the curve of the fluorescent peaks specific for each allele was similar in cDNA and genomic DNA (mean values : 0.98 ± 0.079 and 1.01 ± 0.082, p = 0.8). This indicated that the steady state mRNA level for the mutated allele (A) and the wild allele (G) was not altered.

### 4. Sequence analysis of EDNRB cDNA

The sequence analysis of the EDNRB cDNA was performed in10 patients, heterozygotes for the EDNRB-30G>A polymorphism. No further mutation was detected on the entire coding sequence of the ET_B _receptor gene, ruling out other mutations of the *EDNRB *gene in linkage disequilibrium with the EDNRB-30G>A polymorphism.

## Discussion

The main results of this study are : 1) the 30G>A SNP in *EDNRB *is strongly associated with a low FEV_1 _in a population of adult asthmatic patients; 2) no relationship was found between pulmonary function and *EDNRA *or *preproET-1 *polymorphism; 3) in a large population of adults from the general population, a similar association between low FEV1 and GG genotype was found, with similar results observed in the subgroups of asthmatic and heavy smokers. Altogether, these data emphasize that *EDNRB *polymorphism could be a determinant of airway obstruction in patients with predisposing factors.

As EDN1 displays physiological effects in the airways, the initial purpose of this study was to look for a possible association between FEV_1 _in asthma and the genetic differences in the endothelin system in asthmatic patients. We investigated genetic polymorphisms with high mutated allelic frequency in the general population : the 595G>T polymorphism of the gene coding for the *preproET-1 *(21), the 1363C>T polymorphism of the *EDNRA *gene (22) and the 30G>A polymorphism of the *EDNRB *gene (22). In our asthmatic population, for each polymorphism, the observed genotypes repartition and allelic frequencies were similar to previously reported data obtained in caucasian populations (21,22). These repartitions do not support an association between these polymorphisms and asthma. In a Czech population, no association between polymorphism for the intron 4 (position 8000) in the *EDN1 *gene and asthma or atopy was observed [33]. In a British and a Japanese population studying other genetic variants of the *preproET-1, EDNRA and EDNRB *genes, no association was observed with asthma (28). The variants of the *EDNRA *gene showed a marginal association with atopy in the British population but no association in the Japanese population of asthmatics. We cannot compare our results with the latter study since they did not measure pulmonary function (28).

Our asthmatic patients were exclusively recruited from our university hospital, leading probably to a selection bias of more severe patients. However, we have no argument to support that this could influence the relationship between the *EDNRB *gene polymorphism and the degree of airway obstruction assessed by FEV_1_. To finish with, since we did not genotype any random markers, we cannot completely rule out population stratification as an explanation for our results.

In the hospital-based asthmatic population, we found a strong association between the EDNRB-30G>A SNP and the degree of airway obstruction. Indeed, FEV_1 _values were lower in GG patients than in AA+AG patients, and tended to be lower in AG patients than in AA patients. Most importantly, in a population-based sample of subjects recruited as part of the ECRHS, we observed that the percentage of subjects with FEV1 % predicted below 80% was higher in GG subjects than in AG+AA subjects. As we analysed the subgroup of asthmatic subjects in this population, we again found a strong association between the EDNRB-30G>A SNP and the degree of airway obstruction. Interestingly, a similar correlation was observed in the subgroup of smokers, whereas when focusing on never smokers, such a correlation was not observed. Our results suggest that the GG genotype may contribute to the development of airway obstruction in subjects with predisposing conditions, such as asthmatic inflammation or tobacco exposure. Bronchial hyperresponsiveness has been associated with the long term development of airway obstruction in asthma and in smokers [34]. However, in the present study, the *EDNRB *polymorphism did not influence the prevalence of bronchial hyperresponsiveness in the ECRHS group (data not shown).

To date, the mechanism of the association between *EDNRB *genotype and FEV_1 _is unknown. Numerous studies have demonstrated the role of EDNR1 on bronchial smooth muscle contraction (*via *intracellular calcium input and modulation of acetylcholine or relaxant substances such as prostanoids or nitric oxide secretion), but also on mucus secretion, airway remodelling and inflammation, all features contributing to airway obstruction in asthmatics and smokers [35]. Since the EDNRB 30G>A polymorphism is a silent mutation (22) that does not modify the amino acid sequence of the protein, we imagined that this mutation could modify the transcription of the gene and/or the stability of the mRNA either directly or by a linkage disequilibrium with a mutation located in the regulating region of the gene. To investigate this point, we performed a relative quantification of mRNAs transcribed from each allele. We found no allelic difference in the mRNA level in heterozygote patients for the EDNRB-30G>A polymorphism. Thus, we can suggest that this polymorphism is unlikely to modify the expression of the EDNRB mRNA. We could then hypothetise that the EDNRB-30G>A polymorphism is in linkage disequilibrium with a non silent mutation located in the coding region of the gene. However, we found no difference in the nucleotide sequence from G and A alleles of the EDNRB cDNA from 10 heterozygote patients, making unlikely this possibility. However, we cannot rule out that the EDNRB-30G>A polymorphism may be in linkage disequilibrium with a mutation located in another gene near to the EDNRB locus in 13q22, a previously described region as an asthma susceptibility locus (29). Further studies are under way to explore this hypothesis.

## Conclusion

In summary, the present study is the first report for a strong association between the EDNRB-30G>A polymorphism and the degree of airway obstruction, especially in patients with factors predisposing to airway remodelling such as asthma or smoking. Further studies, including complete sequencing of the EDNRB gene and comprehensive assessment of it in other populations, are clearly needed, to confirm this association in asthmatic patients, to determine whether this association exists in other airway obstructive disease such as COPD and to understand its biological pathways.

## Competing interests

The author(s) declare that they have no competing interests.

## Authors' contributions

CT wrote the manuscript and collected data from the hospital-based population of asthmatic patients. AG, BL and FN are strongly involved in the ECRHS study. They collected data, performed statistical analysis of data issued from the ECRHS and wrote a part of the manuscript. AA and JB collected data from the hospital-based population of asthmatic patients.

MP and MA collected data from asthmatic patients, obtained informed consent for genotyping and performed statistical analysis.

JB and SV genotyped all the patients and performed molecular biology experiments.

BC designed the study, collected patients consent and drafted the manuscript.

All authors read and approved the final manuscript.

## Pre-publication history

The pre-publication history for this paper can be accessed here:


